# Prolonged activation of cytomegalovirus early gene *e1*-promoter exclusively in neurons during infection of the developing cerebrum

**DOI:** 10.1186/s40478-021-01139-0

**Published:** 2021-03-09

**Authors:** Isao Kosugi, Yoshifumi Arai, Satoshi Baba, Hideya Kawasaki, Toshihide Iwashita, Yoshihiro Tsutsui

**Affiliations:** 1 Department of Regenerative & Infectious Pathology, Hamamatsu University School of Medicine, 1-20-1 Handayama, Higashi-Ku, Hamamatsu, 431-3192 Japan; 2 Department of Diagnostic Pathology, Toyohashi Municipal Hospital, 50 Hachiken-Nishi, Aotake-Cho, Toyohashi, 441-8570 Japan; 3 Department of Diagnostic Pathology, Hamamatsu University School of Medicine, 1-20-1 Handayama, Higashi-Ku, Hamamatsu, 431-3192 Japan; 4 Institute for NanoSuit Research, Hamamatsu University School of Medicine, 1-20-1 Handayama, Higashi-Ku, Hamamatsu, 431-3192 Japan; 5 Faculty of Health Science, Tokoha University, 1230 Miyakoda-Cho, Kita-Ku, Hamamatsu, 431-2102 Japan

**Keywords:** Cytomegalovirus, *e1*-promoter, Congenital infection, Persistent infection, Cerebral neuron, Neurodevelopmental disorder

## Abstract

The brain is the major target of congenital cytomegalovirus (CMV) infection. It is possible that neuron disorder in the developing brain is a critical factor in the development of neuropsychiatric diseases in later life. Previous studies using mouse model of murine CMV (MCMV) infection demonstrated that the viral early antigen (E1 as a product of *e1* gene) persists in the postnatal neurons of the hippocampus (HP) and cerebral cortex (CX) after the disappearance of lytic infection from non-neuronal cells in the periventricular (PV) region. Furthermore, neuron-specific activation of the MCMV-*e1*-promoter (*e1-pro*) was found in the cerebrum of transgenic mice carrying the *e1-pro*-*lacZ* reporter construct. In this study, in order to elucidate the mechanisms of *e1-pro* activation in cerebral neurons during actual MCMV infection, we have generated the recombinant MCMV (rMCMV) carrying long *e1-pro*1373- or short *e1-pro*448-EGFP reporter constructs. The length of the former, 1373 nucleotides (nt), is similar to that of transgenic mice. rMCMVs and wild type MCMV did not significantly differed in terms of viral replication or E1 expression. rMCMV-infected mouse embryonic fibroblasts showed lytic infection and activation of both promoters, while virus-infected cerebral neurons in primary neuronal cultures demonstrated the non-lytic and persistent infection as well as the activation of *e1-pro*-1373, but not -448. In the rMCMV-infected postnatal cerebrum, lytic infection and the activation of both promoters were found in non-neuronal cells of the PV region until postnatal 8 days (P8), but these disappeared at P12, while the activation of *e1-pro*-1373, but not -448 appeared in HP and CX neurons at P8 and were prolonged exclusively in these neurons at P12, with preservation of the neuronal morphology. Therefore, *e1-pro*-448 is sufficient to activate E1 expression in non-neuronal cells, however, the upstream sequence from nt -449 to -1373 in *e1-pro*-1373 is supposed to work as an enhancer necessary for the neuron-specific activation of *e1-pro*, particularly around the second postnatal week. This unique activation of *e1-pro* in developing cerebral neurons may be an important factor in the neurodevelopmental disorders induced by congenital CMV infection.

**Supplementary Information:**

The online version contains supplementary material available at 10.1186/s40478-021-01139-0.

## Introduction

Cytomegalovirus (CMV) is the leading viral cause of developmental brain disorders during the embryonic and perinatal periods in humans [14]. Microcephaly is the most prominent brain-related manifestation of congenital CMV infection [5]. Even if there are no obvious symptoms at birth, the infection occasionally persists and causes neurological disorders, including sensorineural hearing loss, mental retardation and, possibly, psychotic diseases [15, 17, 22]. A neuron disorder in the developing brain may be a critical factor in the development of neuropsychiatric diseases in later life, although the mechanism of CMV pathogenesis in the developing brain remains poorly understood.

Although studies on human subjects, particularly in vivo studies, have obvious limitations, and while CMV has strict species specificities, animal models using murine CMV (MCMV) in place of human CMV (HCMV) have been undertaken [49, 55, 59]. MCMV shows similar characteristics to HCMV in terms of cell tropism, pathology and immunity in host–pathogen interactions [42]. Furthermore, the similarity of the MCMV genome to the HCMV genome at the genetic and nucleotide composition levels [41] and of the gene expression pattern of three sequential phases; immediate-early (IE), early (E) and late (L), have been reported [35]. In addition, as many developmental processes in mice continue to occur during the postnatal period [16, 62], murine neonates are useful in the investigation of the effects of infection on brain development.

The susceptibility of the brain cells and neuropathological findings are considered to be related to the developmental stages of the brain at which congenital CMV infection occurs [55]. In the early phase of brain development, neural stem/progenitor cells (NSPCs) have been reported to be the major target cells for MCMV and HCMV in the periventricular (PV) region including ventricular and subventricular zone [31, 40, 44, 51]. Most of the infected cells in this area are fully permissive for viral replication and show lytic infection, resulting in disruption of NSPC proliferation and differentiation [28, 37, 44]. During postnatal brain development in mice, a unique persistent infection occurs exclusively in the developing neurons of the hippocampus (HP) and cerebral cortex (CX). This infection was first presented by immunostaining for the MCMV early nuclear antigen (E1) [53, 54]. MCMV-E1 as the product of the MCMV *e1* gene (M112-113) is well-known to corresponds to the product of the HCMV *e1* gene (UL112-113) [10, 63]. In the developing brains of newborn mice infected with MCMV, the expression of the E1 antigen first appears in immature neural cells and non-neural cells in the PV region, and thereafter the expression of E1 disappears from the PV region but is retained exclusively in neurons of the HP and CX then up to 1 month after birth [29, 52].

Previously we generated transgenic mice carrying the *e1-*promoter (*e1-pro*) connected with reporter gene *lacZ* [3]. These transgenic mice demonstrated neuron-specific activation of *e1-pro*, particularly after birth, suggesting that a differential transcriptional regulation of *e1* gene in neurons may support the neuron-specific infectious dynamics and which appears to be related with the neurological disorders induced by CMV. However, MCMV-E1 [33, 46], as well as HCMV-E1 [1, 47], is essential for viral DNA replication and transcriptional regulation, and is expressed in all cells permissive for viral infection. Previous studies concerning the transcriptional regulation of *e1* gene, focused on the *e1-pro* sequence spanning the region from the transcriptional start site up to ~ 300 nucleotides (nt) [4, 39, 47]. Furthermore, those experiments were performed using fibroblasts or non-neuronal cell lines. On the contrary, we have focused on the long *e1-pro* sequence of ~ nt 1400 and our transgenic mice demonstrated the differential transcriptional activation of *e1-pro* in developing cerebral neurons, the activation of which was enhanced by MCMV infection. Nevertheless, there are fundamental differences in terms of *e1-pro* activation between transgenic mice and actual MCMV infection as the *e1-pro* sequence exists on the host genome in the former but on the viral genome in the latter. Thus, further investigation is needed to determine whether the long *e1-pro* on the viral genome is similarly activated during actual CMV infection of the developing neurons as in the transgenic mice. Furthermore, the differences in the mechanisms of *e1-pro* activation between neurons and non-neuronal cells still remains to be clarified.

In this study, in order to investigate the spaciotemporal activation of *e1-pro* on the viral genome, we have constructed the recombinant MCMV (rMCMV) expressing EGFP as a reporter under the control of the long (nt 1373) or truncated short (nt 448) *e1-pro* fragment, rMCMV1373 or rMCMV448, respectively. We then performed in situ detection of *e1-pro* activity during rMCMV infection of primary neuronal cultures and neonatal mouse brains. Infection with rMCMV1373 demonstrated neuron-specific activation of MCMV *e1-pro* in both primary neuronal cultures and cerebral neurons, particularly around in the second postnatal week. However, infection with rMCMV448 did not show the neuronal activation of MCMV *e1-pro* in either model. Our results indicated that the upstream region from nt -449 to -1373 in the long *e1-pro* sequence is necessary for prolonged *e1-pro* activation in the developing cerebral neurons.

## Materials and methods

### Mice

Inbred specific pathogen-free pregnant ICR mice were obtained from SLC Japan (Hamamatsu, Japan). Newborn mice were maintained by maternal suckling during the postnatal period. All experimental procedures were performed in accordance with “Fundamental Guidelines for Proper Conduct of Animal Experiment and Related Activities in Academic Research Institutions under the jurisdiction of the Ministry of Education, Culture, Sports, Science and Technology (Ministry of Education, Culture, Sports, Science and Technology, Notice No. 71, 2006)” and “Guidelines for Proper Conduct of Animal Experiments (The Guide; Science Council of Japan, 2006)”, and were approved by the Animal Care Committee of Hamamatsu University School of Medicine (#2011057). Viral inoculation and sacrifice were performed under anesthesia, and all efforts were made to minimize suffering.

### Virus and plaque assay

The Smith strain of wild type MCMV, which had been passaged in mouse embryonic fibroblasts (MEFs), was provided by Dr. Y. Minamishima (Miyazaki, Japan, [21]). MEFs were prepared from 12.5-day-old embryos of BALB/c mice (SLC Japan), and were grown in Dulbecco's modified Eagle's essential medium (DMEM; Sigma-Aldrich, Merck, #D5796, Darmstadt, German) containing penicillin (100 units /ml), streptomycin (50 μg/ml), and 10% fetal bovine serum (FBS; Gibco, Thermo Fisher Scientific, #26140079, Waltham, MA). The viral titer was determined by a plaque assay in MEF monolayers as described previously [61].

### Recombinant viruses

**Fig. 1:**
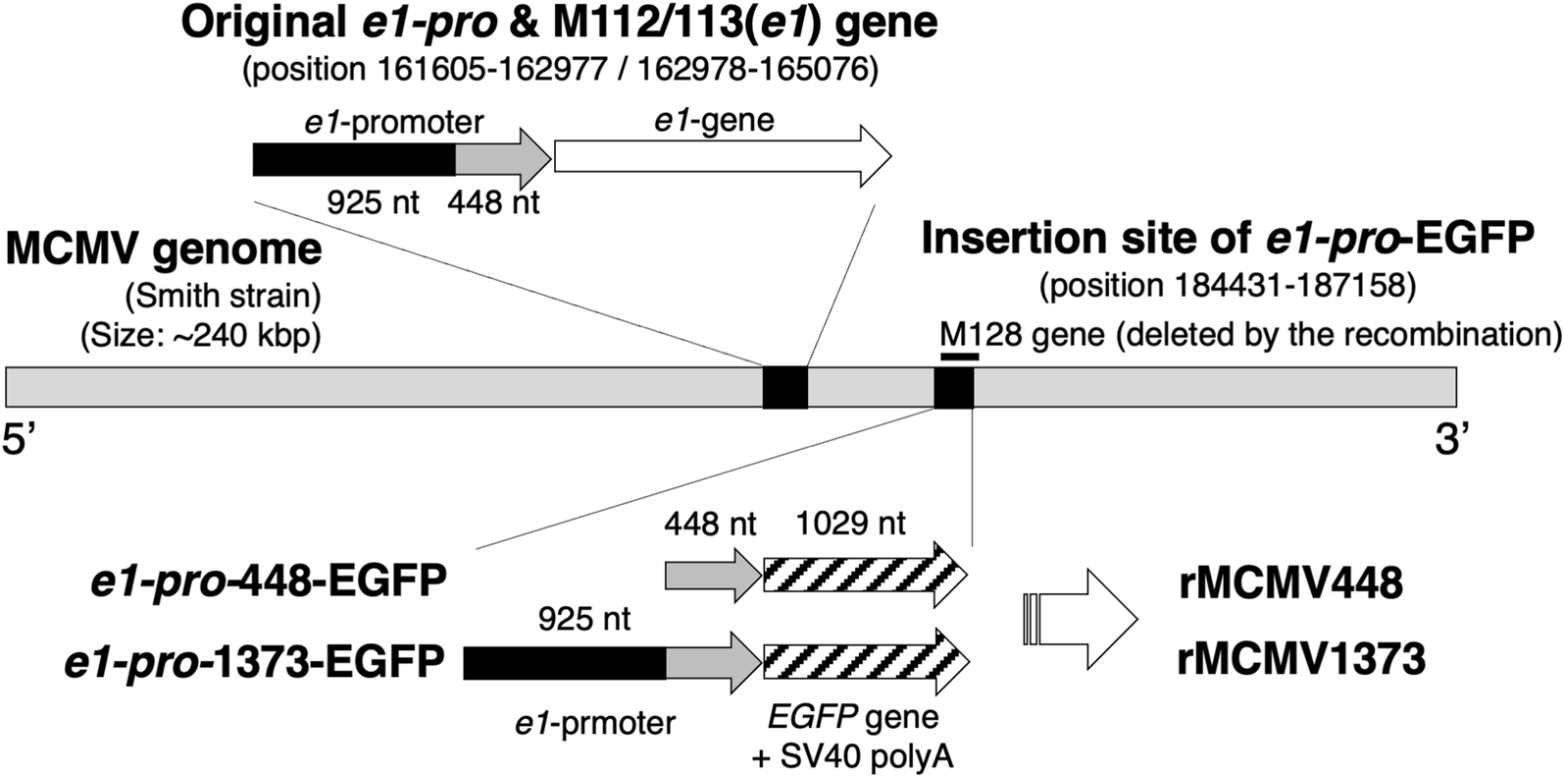
Constructions of rMCMV448 and rMCMV1373. The arrangement of the original MCMV-*e1-pro* (position nt 161605 to 162977, black and gray arrow), M112/113 (*e1*) gene (position nt 162978 to 165076, white arrow) and the insertion site of *e1-pro*-EGFP (right black box) in the wild type MCMV genome are shown. In this study, an *e1-pro*-EGFP cassette consisting of the *e1-pro* fragment (nt -1373 or -448 to + 38 relative to the transcription start site, black and gray or gray arrow, respectively) and EGFP gene with an SV40-derived polyadenylation signal (striped arrows) was inserted into the position between nt 184431 and 187158 (right black box) in wild type MCMV genome by homologous recombination. This recombination causes the deletion of the nt 2728 sequence including the greater part of the M128 gene (black line). During the infection of recombinant viruses, the activation of the inserted *e1-pro* can be in situ detected as the expression of EGFP. The detailed construction, preparation method and verification of rMCMVs are shown in Additional file 1: Fig. S1

Recombinant viruses (rMCMV1373 and rMCMV448) derived from the Smith strain of wild type MCMV (Gene accession number U68299) capable of expressing enhanced green fluorescent protein (EGFP; Gene accession number U55763, Clontech, Takara Bio USA, Mountain View, CA) as a reporter were used in this study (Fig. 1). Both viruses were constructed to express an EGFP gene insert under control of the MCMV *e1-pro* fragment. The *e1-pro* fragment of rMCMV1373 or rMCMV448 was derived from a DNA sequence from the transcription start site (position 162978) to nt -1373 (position 161605) or to nt -448 (position 162530), respectively. An *e1-pro*-EGFP cassette, which consisted of the *e1-pro* fragment including a sequence from nt -1373 or -448 to + 38 relative to the transcription start site, EGFP gene and SV40-derived polyadenylation signal, was inserted into the position between 184431 and 187158 in wild type MCMV genome by homologous recombination. According to the method for the construction of rMCMV-MC.55 as reported by Dr. Mocarski’s laboratory [58], we determined the insertion site of our rMCMVs as the same site as rMCMV-MC.55. This recombination causes the deletion of the nt 2728 sequence including the greater part (exon 1, exon 2 and most of exon 3) of the MCMV M128 gene (position from 186085 to 187296). However, M128 gene is completely dispensable for viral growth in cell culture as well as for growth, latency, and pathogenesis in mice [11]. It is supposed that the deletion of the M128 gene has almost no effect on endogenous *ie* promoter activation. The detailed construction, preparation method and verification of rMCMVs are shown in Additional file 1: Fig. S1.

### Primary neuronal culture

Primary neuronal cultures were prepared from the CX and HP of gestational day 16 embryos of ICR mice with a minor modification of the method as described in a previous study [27]. Brains were removed from the embryos and put in a plastic dish on ice. The CX and HP were separated from the diencephalon and meninges under a compound microscope and scissored in neurobasal medium (Gibco, #A2477501). After digestion with 0.125% trypsin (Gibco, #27250018) in calcium, magnesium-free PBS and 30 mM glucose at 30 °C for 30 min, the cells were suspended and dissociated in the plating medium as described below and plated on 12-well plates or coverslips (18 × 12 mm) in 12-well plates, previously coated with poly-d-lysine (0.1 mg/ml) (Sigma-Aldrich, #P7280). The cells were grown in the plating medium consisting of neurobasal medium supplemented with 10% horse serum (Gibco, #16050130), 1% B27 supplement (Gibco, #17504044), 0.5 mM glutamine, 50 μM 2-mercaptoethanohol, penicillin (100 units /ml) and streptomycin (50 μg/ml). Two days after plating, the cells were treated with 5-fluoro-2′-deoxyuridine (0.04 mM) (Sigma-Aldrich, #F0503) for 2 days to inhibit proliferation of non-neuronal cells, and then the medium was refreshed and not changed again. We used primary neuronal cultures for experiments from 9 days after plating. In this study, our primary neuronal culture was predominantly composed of neurons, but certainly contained some non-neuronal cells, neurons, astrocytes and other cells present at an approximate ratio of 90:5:5.

### MCMV infection

MEF and primary neuronal cultures at 2 days or 9 days after plating, respectively, were infected with MCMVs at different multiplicities of infection (MOIs) as follows: MEF at 0.1, 0.3 or 1 plaque forming units (PFU)/cell and primary neuronal cultures at 0.3, 1 or 3 PFU/cell. To determine the time courses of viral titers, MEF and primary neuronal cultures infected with MCMVs at MOIs of 1 and 3, respectively, were used. At different numbers of days post-infection (dpi), three 50-µl samples of culture supernatant in each MCMV-infected group were collected. The viral titers were measured by a plaque assay. MEF and primary neuronal cultures on glass coverslips were fixed at 2 and 4 days after the infection, respectively, by 4% paraformaldehyde (PFA) in 0.1 M phosphate buffer (PB) for 20 min at room temperature, stored in PBS with 5% FBS and 0.1% sodium azide at 4 °C, and followed by the immunofluorescence.

For the infection of the neonatal mouse brain, 1 day after birth, 4 × 10^4^ PFU of MCMVs in 5 µl of minimum essential medium (MEM) was injected into the right cerebral hemisphere of neonatal mice under cryoanesthesia using a 10-μl Hamilton syringe with a 27-gauge needle from the midpoint between the ear and eye. At 7 or 11 dpi, mice were killed under anesthesia with ether. The brains were removed, stored at –80 °C and used for plaque assays or reverse transcription-polymerase chain reaction (RT-PCR) assays. At 3, 7 or 11 dpi, the brains for histological analysis were fixed by perfusion with 4% PFA, removed, fixed for 2 days at 4 ºC, immersed in 30% sucrose for 2 days and quickly frozen in *n*-hexane at − 80 °C. Coronal sections used for the detection of EGFP fluorescence and immunofluorescence were cut at 60 µm using a cryostat and stored in PBS with 5% FBS and 0.1% sodium azide at 4 °C, while the sections for the immunohistochemistry were cut at 10 µm, air-dried and stored at –80 °C.

### Immunofluorescence, immunohistochemistry and cell counts

MCMV-E1 nuclear antigen (Ag) was detected by mouse monoclonal antibody (mAb) clone D5 (IgG2a; 1:1, [24, 54]). MCMV-IE3 nuclear Ag was detected by rat polyclonal Ab (pAb; 1:1000, [24]). MCMV-M45 cytoplasmic Ag was detected by rat mAb clone Q3 (1:1, [27]).

For immunofluorescence, in addition to MCMV-E1 or -M45, neurons were stained by Ab specific for NeuN (mouse mAb, clone A60, IgG1, 1:500, Millipore, Merck, #MAB377) or MAP2 (rabbit pAb, 1:100, abcam, #ab32454 Cambridge, UK). Astrocytes were stained by rabbit pAb specific for GFAP (1:1, DAKO, Agilent, #GA524, Santa Clara, CA). The following secondary antibodies, purchased from Invitrogen, Thermo Fisher Scientific, were used at a 1:500 dilution; Alexa Fluor 546 anti-mouse IgG2a (#A-21133), anti-mouse IgG1 (#A-21123), -rabbit IgG (#A-11035) or -rat IgG (#A-11081). Nuclei were stained with 4′,6-diamidino-2-phenylindole, dihydrochloride (DAPI) (Sigma-Aldrich, #D9542). Immunostained cells on coverslips or sections were mounted with ProLong Gold antifade reagent (Invitrogen, #P36934).

For immunohistochemistry, after microwave treatment, brain sections were reacted with anti-E1 mouse mAb D5, anti-IE3 rat pAb, anti-M45 rat mAb Q3 or anti-EGFP rabbit pAb (1:100, abcam, #ab6556). The sections, except for those reacted with mAb D5, were subsequently reacted with horseradish peroxidase (HRP)-conjugated goat anti-rat IgG pAb (N-Histofine simple stain mouse MAX-PO(Rat); 1:1, Nichirei Bioscience, #414311, Tokyo, Japan) or HRP-conjugated goat anti-rabbit IgG (N-Histofine simple stain mouse MAX-PO(R); 1:1, Nichirei Bioscience, #414341). The sections reacted with mAb D5 were reacted with Alexa Fluor 546 goat anti-mouse IgG2a (1:500) and then with HRP-conjugated rabbit anti-goat IgG (N-Histofine simple stain mouse MAX-PO(G); 1:1, Nichirei Bioscience, #414331). The sections were colored with 3,3-diaminobenzidine tetrahydrochloride (DAB; DAKO, #K3467). Nuclei were counterstained with hematoxylin.

Phase contrast and immunofluorescence images (Figs. 3, 4) were taken using an Olympus IX-70 fluorescence microscope with a DP-70 digital camera system (Olympus, Tokyo, Japan). Cerebral images of EGFP fluorescence (Fig. 6) were taken using a BZ-9000 Biorevo microscope (Keyence, Osaka, Japan). Confocal images were captured using a Leica TCS SP8 microscope with Leica LAS X software (Leica, Wetzlar, German) (Fig. 5) and an Olympus FV1000-D microscope with Fluoview software (Figs. 7, 8). A stack of 5 images from 0.5-µm step slices was combined for the analysis.

At least 3 different samples in each experimental group were used for the analysis. In each coverslip, cell counts from 3 fields (292 × 220 µm; 0.064 mm^2^/a field) were averaged. In the coronal sections of the right hemisphere of rMCMV-infected brains, right hemispheres were divided into the PV, HP, and CX regions and the numbers of EGFP^+^ and MCMV-E1^+^ cells in each area at 3, 7, and 11dpi were counted. Then the numbers of positive cells in the hippocampal and cortical region were combined.

### RT-PCR for the semi-quantification of mRNA expression of MCMV*-ie3, -e1* and EGFP genes in brains infected with wild type MCMV or rMCMVs

Total RNA was extracted from the frozen brain samples using ISOGEN (Wako Chemical, Osaka, Japan), purified with a RNeasy Mini Kit (Qiagen, Hilden, Germany, #74104) and treated with RNase-free DNase I (Qiagen, #79254), according to the manufacturer’s instructions. One hundred ng of cDNA synthesized using a SuperScript III First Strand Synthesis kit with random primers (Invitrogen, Thermo Fisher Scientific, #18080-051) was used for each PCR reaction. The β-actin gene was amplified from each sample as a control. The gene expression levels of the target genes were normalized to the expression levels of the β-actin gene. The primers used for the detection of the indicated mRNAs were as follows: MCMV-*ie3*, 5′-ACG TGG GGA ATG ATA ACA GC-3′ (forward) and 5′- TCC TGA GGC TGC TGA AAA AT-3′ (reverse); MCMV-*e1*, 5′- CCA ACG GTA CCC TTC ATA GG-3′ (forward) and 5′- CAG CTT CGT CTG CAT TAC CA-3′ (reverse); EGFP, 5′-TGA ACC GCA TCG AGC TGA AGG G-3′ (forward) and 5′- TCC AGC AGG ACC ATG TGA TCG C-3′ (reverse); β-actin, 5′-GTG CTA TGT TGC CCT GGA TT-3′ (forward) and 5′-TGG AGT TGA AGG GTG GTC TC-3′ (reverse).

### Statistical analyses

Numerical results were expressed as the mean ± standard error of mean (SEM) obtained from at least three independent experiments. Statistical analysis was performed using an unpaired 2-tailed Student’s t test. Probability values (*p*) < 0.01 were considered to be significant.

## Results

### MCMV *e1-pro* activation during infection in MEF and primary neuronal cultures

#### Growth of wild type MCMV and rMCMVs in MEF and primary neuronal cultures

**Fig. 2:**
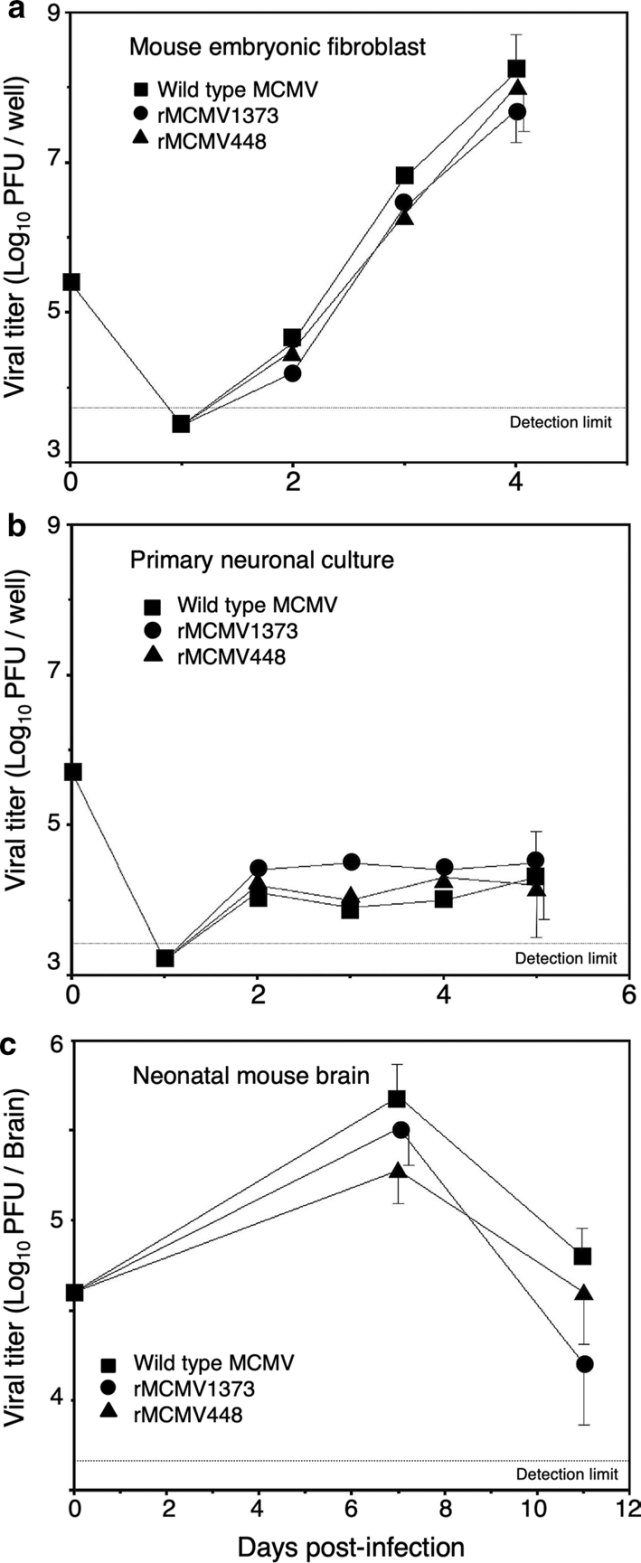
Viral growth in MEFs (**a**), primary neuronal cultures (**b**) and neonatal mouse brains (**c**) infected with wild type MCMV (■), rMCMV1373 (●) or rMCMV448 (▲). MEF and primary neuronal cultures were infected with MCMVs at MOIs of 1 and 3, respectively. Neonatal mice were intracerebrally infected with 4 × 10^4^ PFU of MCMVs at 1 day after birth. Viral loads in each culture supernatant or mouse brain were determined by infectious viral titers. The dotted line indicates the detection limit. Mean ± SEM of 3 samples in infected cultures or mouse brains in each experimental group are shown

To investigate the effects of the recombination of wild type MCMV on in vitro viral replication, we examined the growth of wild type MCMV, rMCMV1373 and rMCMV448 in MEF and primary neuronal cultures. In MEFs, which are known to be fully permissive for MCMV replication, all viruses showed rapid exponential growth (Fig. 2a). In primary neuronal cultures, the growth of all viruses was very low and prolonged, and showed persistent infection as compared with the growth in MEFs (Fig. 2b). However, in both cultures there were no significant differences in the titers at any time point among three viruses. Therefore, it was considered that the recombination of wild type MCMV in this study had almost no effect on viral replication.

#### No difference in the activation of the MCMV e1-promoter between rMCMV-1373 and -448 infected MEFs

**Fig. 3:**
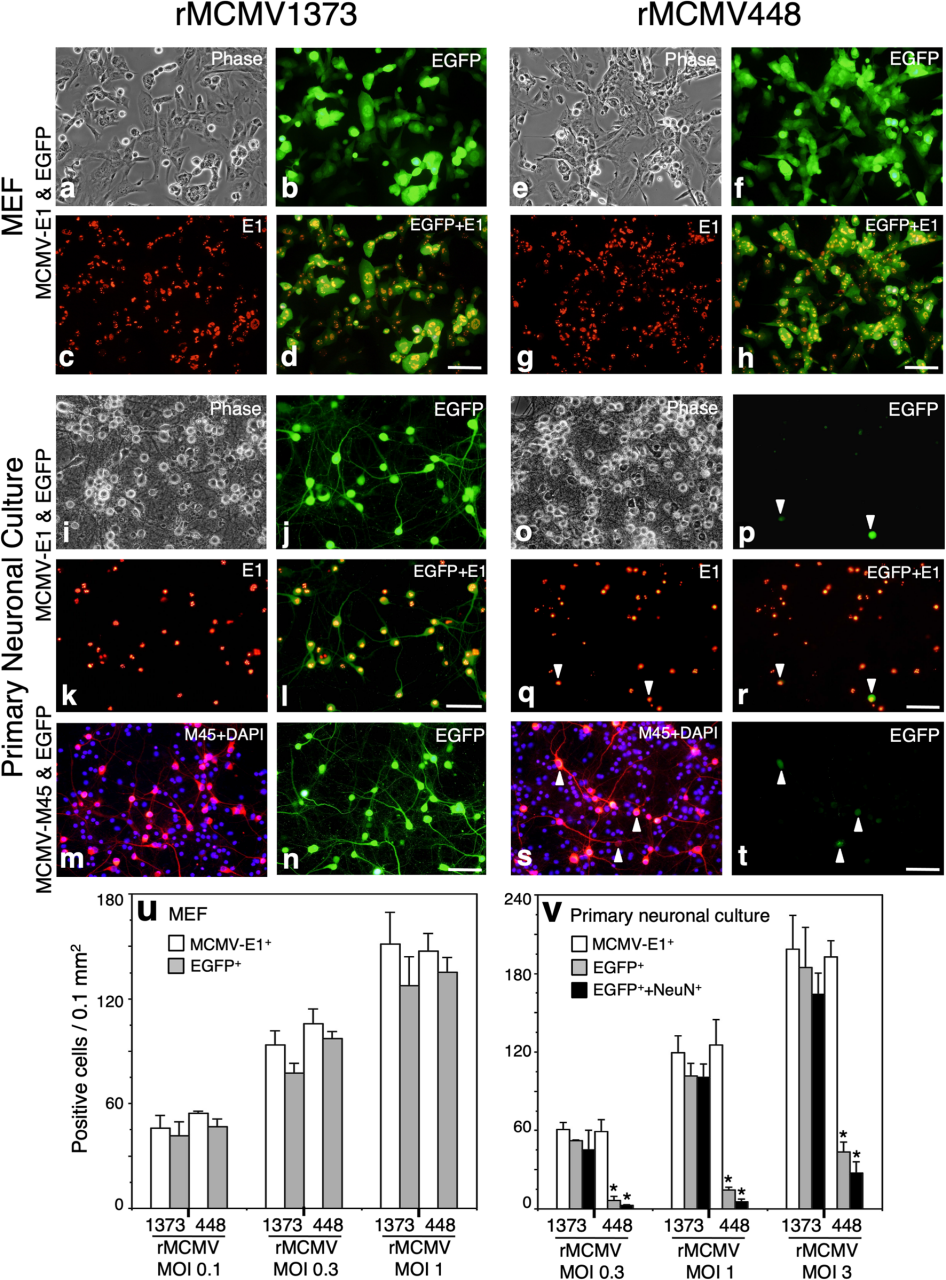
**a**–**t** Detection of EGFP, MCMV-E1 and MCMV-M45 in rMCMV1373- or 448-infected MEF and primary neuronal cultures. Either rMCMV was allowed to infect both cell cultures at a MOI of 1. Virus-infected MEF and primary neuronal cultures were sampled at 2 and 4 dpi, respectively. **a**–**d** rMCMV1373-infected MEFs. **e**–**h** rMCMV448-infected MEFs. **i**–**n** rMCMV1373-infected primary neuronal culture. **o**–**t** rMCMV448-infected primary neuronal culture. In each rMCMV-infected culture, phase contrast (**a**, **e**, **i**, **o**) and fluorescence views of EGFP (**b**, **f**, **j**, **n**, **p**, **t**), immunofluorescence for MCMV-E1 (red) (**c**, **g**, **k**, **q**) and MCMV-45 (red) (**m**, **s**) and merged images of EGFP and MCMV-E1 (**d**, **h**, **l**, **r**) are shown. Arrow heads in **p**–**t** indicate EGFP^+^ cells. Scale bar: 50 µm. **u**, **v** Comparison of the numbers of EGFP^+^- or MCMV-E1^+^ cells in MEF (**u**) and primary neuronal cultures (**v**) infected with rMCMV1373 or rMCMV448. MEF and primary neuronal cultures were infected with rMCMVs at three different MOIs and analyzed at 2 and 4 dpi, respectively. Additionally, in primary neuronal cultures (**v**) the numbers of cells doubly positive for EGFP and NeuN as shown in Fig. 4 were counted. The cell counts of 3 different fields (292 × 220 µm; 0.064 mm^2^/a field) were averaged in each coverslip. Mean ± SEM of 3 different coverslips in each experimental group are shown (E1^+^, white bar; EGFP^+^, gray bar; EGFP^+/^NeuN^+^, black bar). Averaged numbers of total cells as DAPI^+^ nuclei were 339 ± 31/0.01 mm^2^ and 305 ± 20/0.01 mm^2^, in virus-infected MEF and primary neuronal cultures, respectively. The averaged percentage of NeuN^+^ cells in primary neuronal cultures infected with rMCMVs was 87.6 ± 3.6%. *p < 0.01 vs. corresponding rMCMV1373-infected culture in each experimental group by an unpaired 2-tailed Student’s *t*-test

**Fig. 4:**
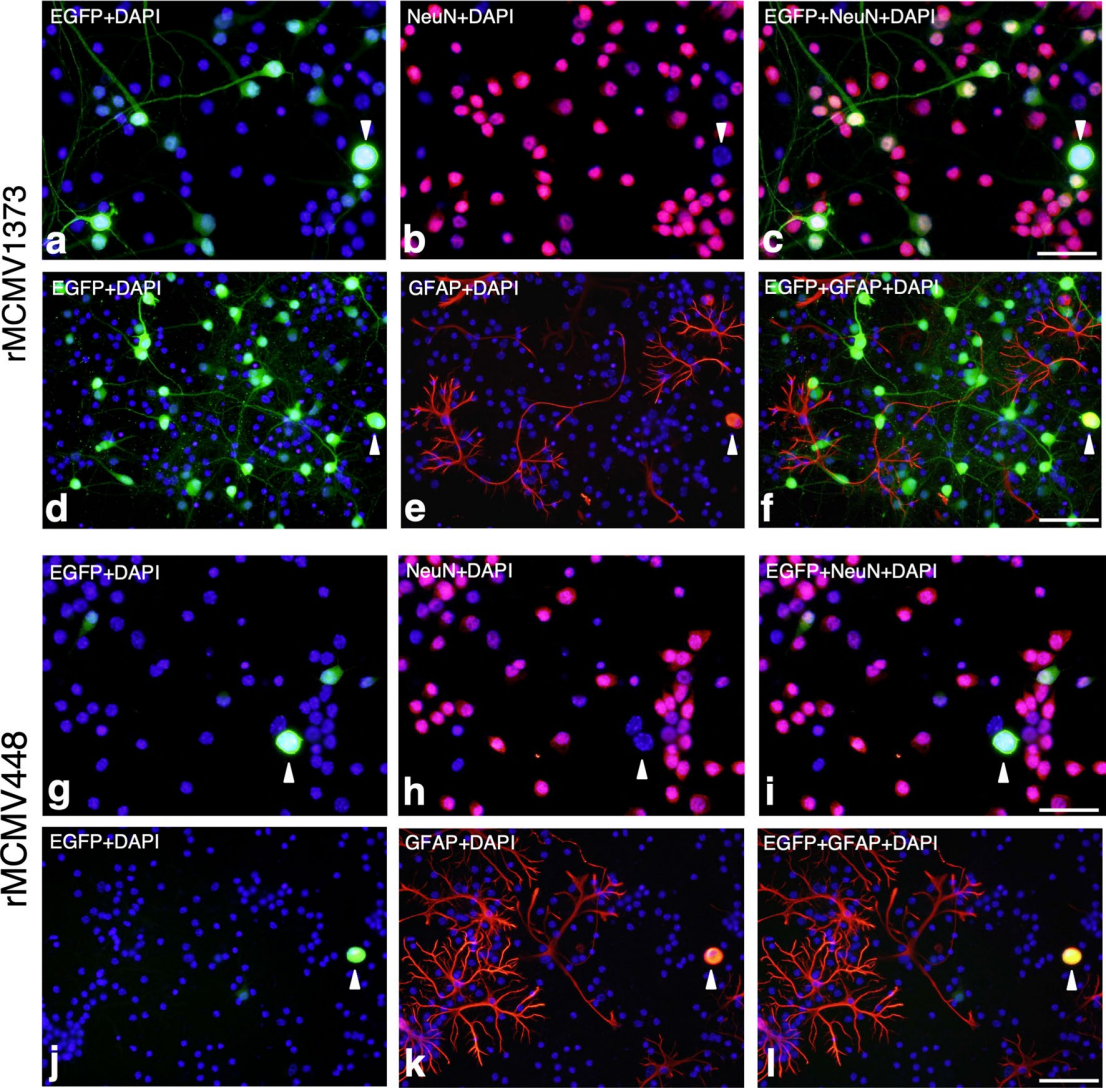
Identification of EGFP^+^ cell types in primary neuronal cultures infected with rMCMV1373 (**a**–**f**) or rMCMV448 (**g**–**l**) as shown in Fig. 3i–t. Immunofluorescence were performed using antibodies against NeuN (**a**–**c**, **g**–**i**) and GFAP (**d**–**f**, **j**–**l**) as neuronal and glial cell makers, respectively. Nuclei are stained with DAPI. In each rMCMV-infected culture, fluorescence views of EGFP (**a**, **d**, **g**, **j**), immunofluorescence for NeuN (purple) (**b**, **h**) and GFAP (red) (**e**, **k**) and merged images of EGFP and cell markers (**c**, **f**, **i**, **l**) are shown. Arrow heads indicate EGFP^+^/NeuN^−^ or EGFP^+^/GFAP^+^ round cells. In rMCMV1373-infected primary neuronal cultures almost all EGFP^+^ cells are neurons (**a**–**c**), while in rMCMV448-infected cultures there are very few EGFP^+^ neurons (**g**–**i**). In both rMCMV1373- and rMCMV448-infected primary neuronal cultures (**f**, **l**), EGFP is not expressed GFAP^+^ differentiated astrocyte with a coral-like shape, though very few EGFP^+^/GFAP^+^ round cells are found. Scale bars: **a**–**c**, **g**–**i** 25 µm; **d**–**f**, **j**–**l** 50 µm

To investigate the difference in MCMV *e1-pro* activation as a reporter of EGFP between non-neuronal cells and neurons, we examined EGFP expression in MEF and primary neuronal cultures infected with rMCMVs (Figs. 3, 4).

In MEFs infected with either rMCMV1373 or rMCMV448 at 2 dpi, increases in MOI led the increases in the numbers of EGFP^+^ cells in parallel with those of E1^+^ cells (Fig. 3u). Virus-infected cells appeared round in shape, indicating a cytopathic effect (CPE) as a sign of the onset of lytic cell death (Fig. 3a, e) and, 4 dpi later, most of the infected cells were detached. There was no significant difference in the numbers of EGFP^+^ or E1^+^cells between rMCMV1373- and rMCMV448-infected MEFs (Fig. 3b, c, f, g, u). At any MOI, the proportion of EGFP^+^ cells to E1^+^ cells was around 90% or more (Fig. 3u), and the immunofluorescence for E1 demonstrated that almost all EGFP^+^ cells expressed E1 in MEFs infected with either rMCMV1373 or rMCMV448 (Fig. 3d, h). These results indicate that the activation of the inserted *e1-pro* in the MCMV genome well represents the actual production of E1-protein, and that the truncation of the *e1-pro*-1373 sequence had no effect on the activation of *e1-pro* in rMCMV-infected MEFs.

#### Distinct neuronal activation of the MCMV e1-promoter in rMCMV1373-infected primary neuronal cultures but not in rMCMV448-infected cultures

In rMCMV-infected primary neuronal cultures at 4 dpi, the averaged percentages of neuronal marker (NeuN)- and glial marker (GFAP)-positive cells were 87.6 ± 3.6% and 4.70 ± 0.15%, respectively. rMCMV-infected primary neuronal cultures scarcely demonstrated a CPE or detachment (Fig. 3i, o), unlike the rMCMV-infected MEFs as described above. In rMCMV1373-infected neuronal cultures, increases in MOI led the increases in the numbers of EGFP^+^ cells in parallel with those of E1^+^ cells, and the proportion of EGFP^+^ cells to E1^+^ cells was about 90%, as observed in rMCMV-infected MEFs (Fig. 3j–l, u). Most of EGFP^+^ cells showed a neuronal morphology; that is, round soma with elongated neurites (Fig. 3j). Immunofluorescence demonstrated that almost all EGFP^+^ cells expressed E1 (Fig. 3k, l). Furthermore, the cells expressing both EGFP and NeuN accounted for at least about 90% of EGFP^+^ cells (Fig. 3v).

Conversely, in the rMCMV448-infected neuronal cultures, the numbers of EGFP^+^ cells were markedly reduced and ranged from 12 to 24% of those in the rMCMV1373-infected neuronal cultures (Fig. 3v), although there was no significant difference in numbers of E1^+^ cells between the rMCMV1373- and rMCMV448-infected neuronal cultures at any MOI (Fig. 3k, q, v). Immunofluorescence revealed the presence of very few EGFP^+^ cells (Fig. 3p, r, t, arrowheads); however, those present did express E1 (Fig. 3q, r, arrowheads), whereas little EGFP was expressed in NeuN-positive neurons in comparison with rMCMV1373-infected neuronal cultures (Fig. 3v). In addition, as observed for the expression of E1, MCMV cytoplasmic antigen M45 was also expressed in almost all the cells with a neuronal morphology in either rMCMV1373- or rMCMV448-infected neuronal culture (Fig. 3m, n, s, t).

Collectively, *e1-pro*-1373 was similarly activated in neurons as in MEFs (Fig. 3b, j), while *e1-pro*-448 was hardly activated in neurons despite adequate activation in MEFs (Fig. 3f, p). However, there were no fundamental differences in the kinetics of viral growth or the expression of viral antigens between rMCMV1373 and rMCMV448 in primary neuronal cultures (Figs. 2b, 3k, m, q, s, v). Therefore, the upstream region from nt -449 to -1373 in the *e1-pro*-1373 sequence is necessary for the neuron-specific activation of *e1-pro*.

#### The MCMV e1-promoter is activated in neurons but not differentiated astrocytes in primary neuronal cultures

To comfirm the distinct neuronal activation of *e1-pro*-1373, immunofluorescence for neuronal and glial markers was performed in rMCMV-infected neuronal cultures. In rMCMV1373-infected neuronal cultures, 90% of EGFP^+^ cells were NeuN^+^ neurons (Figs. 3v, 4a–c). Most of the GFAP^+^ cells were differentiated astrocytes, which displayed a coral-like shape (Fig. 4e, f). Interestingly, EGFP was not expressed in these astrocytes (Fig. 4d–f). In addition, very few cells doubly positive for EGFP and GFAP showed a round shape, suggesting CPE (Fig. 4d–f, arrowheads). On the other hand, in rMCMV448-infected neuronal cultures, there were very few EGFP^+^ cells, and most of them showed a round shape and were doubly positive for GFAP (Fig. 4j–l, arrowheads) but not for NeuN (Fig. 4g–i, arrowheads). These EGFP^+^/GFAP^+^ round cells found in both rMCMV-infected primary neuronal cultures were supposed to be GFAP^+^ immature progenitor or glial cells and permissive for viral replication as with the MEFs as observed above.

**Fig. 5:**
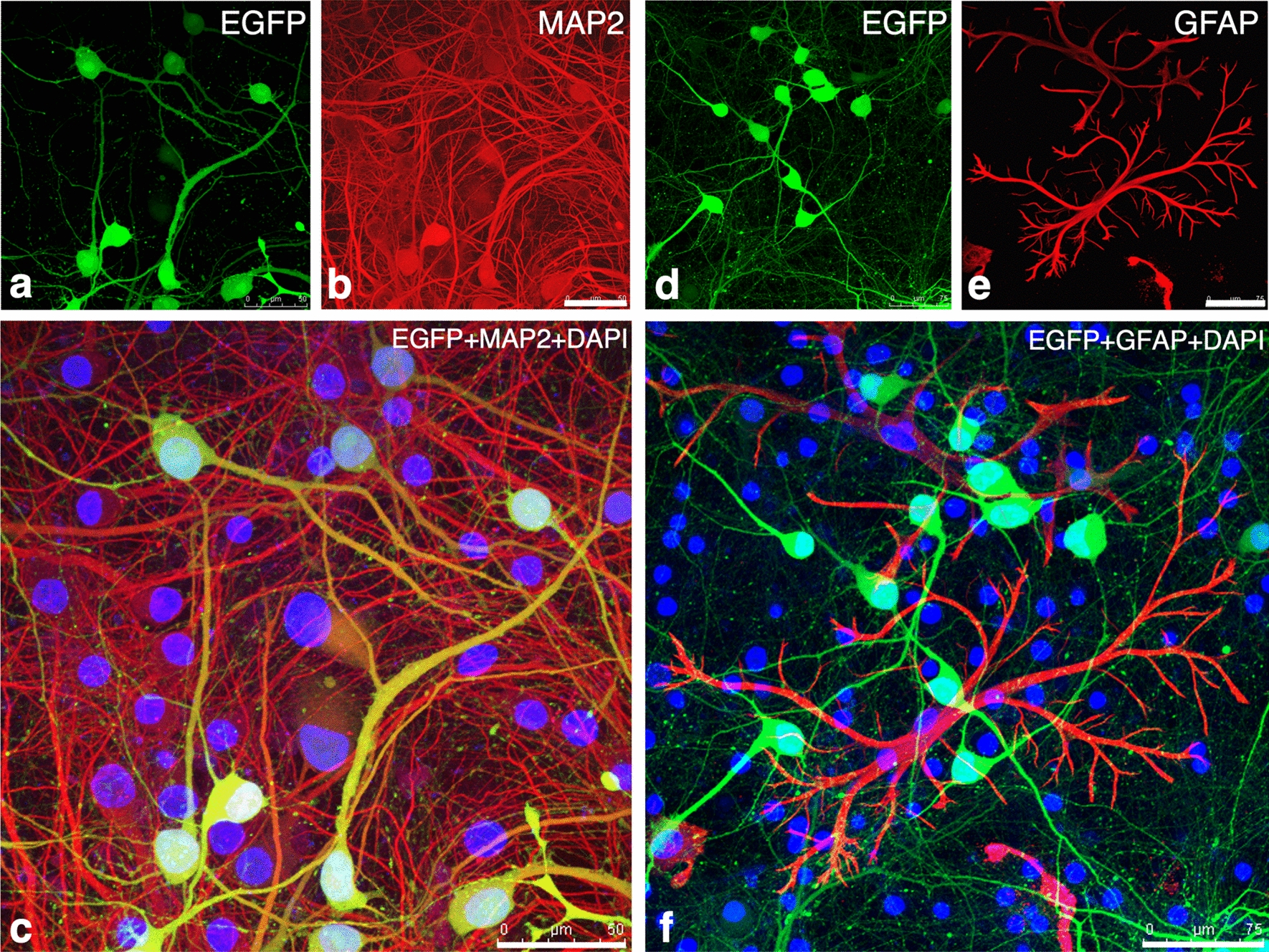
Detailed fluorescence images obtained by confocal laser microscope in rMCMV1373-infected primary neuronal cultures as shown in Fig. 4. The images demonstrate the distinct co-localization of EGFP and the neuronal marker (**a**–**c**) but not the astrocyte marker (**d**–**f**). **a** A fluorescence image of EGFP. **b** Immunofluorescence for MAP2 (red) in the same field as **a**. MAP2 is expressed in numerous primary neuron dendrites and somas. **c** A merged image of **a** and **b**. EGFP^+^ areas appear yellowish because of the co-localization of EGFP and MAP2, indicating that *e1-pro-*1373 is distinctly activated in neurons, as demonstrated in Fig. 4. Nuclei are stained with DAPI. **d** Another fluorescence image of EGFP. **e** Immunofluorescence for GFAP (red) in the same field as **d**. GFAP is expressed in differentiated astrocytes with a coral-like shape, as demonstrated in Fig. 4. **f** A merged image of **d** and **e**. EGFP is not co-localized with GFAP, indicating that the activation of *e1-pro-*1373 does not occur in differentiated astrocytes. Nuclei are stained with DAPI. Scale bars: **a**–**c** 50 µm; **d**–**f** 75 µm

An examination of the immunofluorescence for rMCMV1373-infected neurons using an antibody against MAP2 as another neuronal marker demonstrated that EGFP was accurately co-localized with MAP2 (Fig. 5a–c) based on detailed fluorescent images obtained by confocal laser microscopy. On the contrary, EGFP was not expressed in GFAP^+^ astrocytes (Fig. 5d–f).

From these results, we suppose that the activation of *e1-pro*-1373 but not *e1-pro*-448 is indispensable for the production of the E1-protein in primary neurons. In addition, differentiated primary astrocytes are not permissive in terms of MCMV infection in vitro.

### MCMV-*e1*-promoter activation during infection of neonatal mouse brains

#### Growth of wild type MCMV and rMCMVs in the postnatal brains

To investigate the effects of the recombination of wild type MCMV on in vivo viral replication, we examined the growth of wild type MCMV and rMCMVs in neonatal mouse brains. The intracerebral viral growth peaked around 7 dpi in the ICR mice inoculated with any of the three viruses (Fig. 2c) as reported previously [29]. There were no significant differences in the intracerebral titers at any time point among three viruses, although the viral titer of wild type MCMV was slightly higher than that of rMCMVs. These results indicate that the recombination of wild type MCMV in this study had no significant effect on viral replication and pathogenicity as observed in MEF and primary neuronal cultures.

#### Comparison of the kinetics of the MCMV e1-promorter activity and MCMV-E1-positive cells in the developing brains infected with rMCMV1373 and rMCMV448

**Fig. 6:**
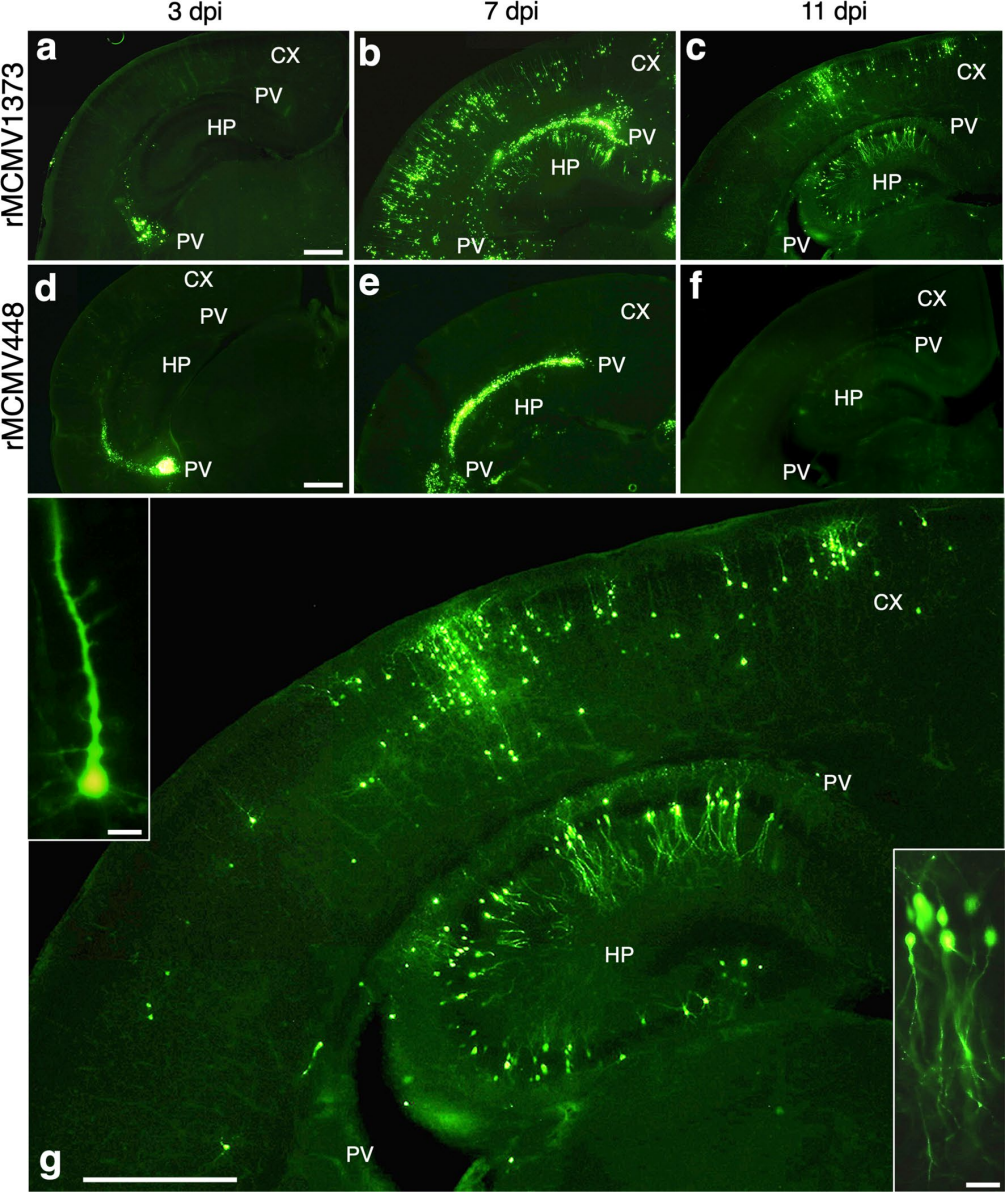
Comparison of MCMV *e1-pro* activity as a reporter of EGFP in neonatal mouse cerebrums infected with rMCMV1373 (**a**–**c**, **g**) or rMCMV448 (**d**–**f**) at 3, 7 and 11 dpi. **a**, **d** At 3 dpi in both the rMCMV1373- and rMCMV448-infected cerebrums, EGFP fluorescence first appears in the PV. **b**, **e** At 7 dpi in rMCMV1373-infected cerebrum, the region of EGFP fluorescence extends from the PV to the HP and CX, while in the rMCMV488-infected cerebrum the region of the fluorescence remains limited to the PV. **c**,** f** At 11 dpi in the rMCMV1373-infected cerebrum, EGFP fluorescence remains in the HP and CX, but disappears in the PV, while in the rMCMV488-infected cerebrum the fluorescence disappears entirely. **g** Enlarged image of **c**. EGFP^+^ cells which display a morphology of pyramidal neurons in the CX (upper left inner panel) and the HP (lower right inner panel). CX, cortex; HP, hippocampus; PV, periventricular region. Scale bars: **a**–**g** 400 µm; **g**-inner panels 80 µm

In the developing cerebrums of neonatal mice infected with either rMCMV1373 or rMCMV448, MCMV *e1-pro* activity as a reporter of EGFP first appeared in the PV region (Fig. 6a, d). At 7 dpi in the rMCMV1373-infected cerebrum the region of EGFP^+^ cells extended from the PV region to the HP and CX (Fig. 6b), while in the rMCMV448-infected cerebrum the region of EGFP^+^ cells still limited to the PV region, (Fig. 6e). At 11 dpi in rMCMV1373-infected cerebrum EGFP^+^ cells continued to remain in the HP and CX although EGFP^+^ cells disappeared from the PV region (Fig. 6c, g). Almost all of the remaining cells in the HP or CX displayed a morphology of the pyramidal neuron. Detailed images of these EGFP^+^ cells showed neurons with neuronal somas and dendrites (Fig. 6g, inner panels). However, in the rMCMV448-infected cerebrum at 11 dpi, the EGFP fluorescence disappeared entirely (Fig. 6f).

**Fig. 7:**
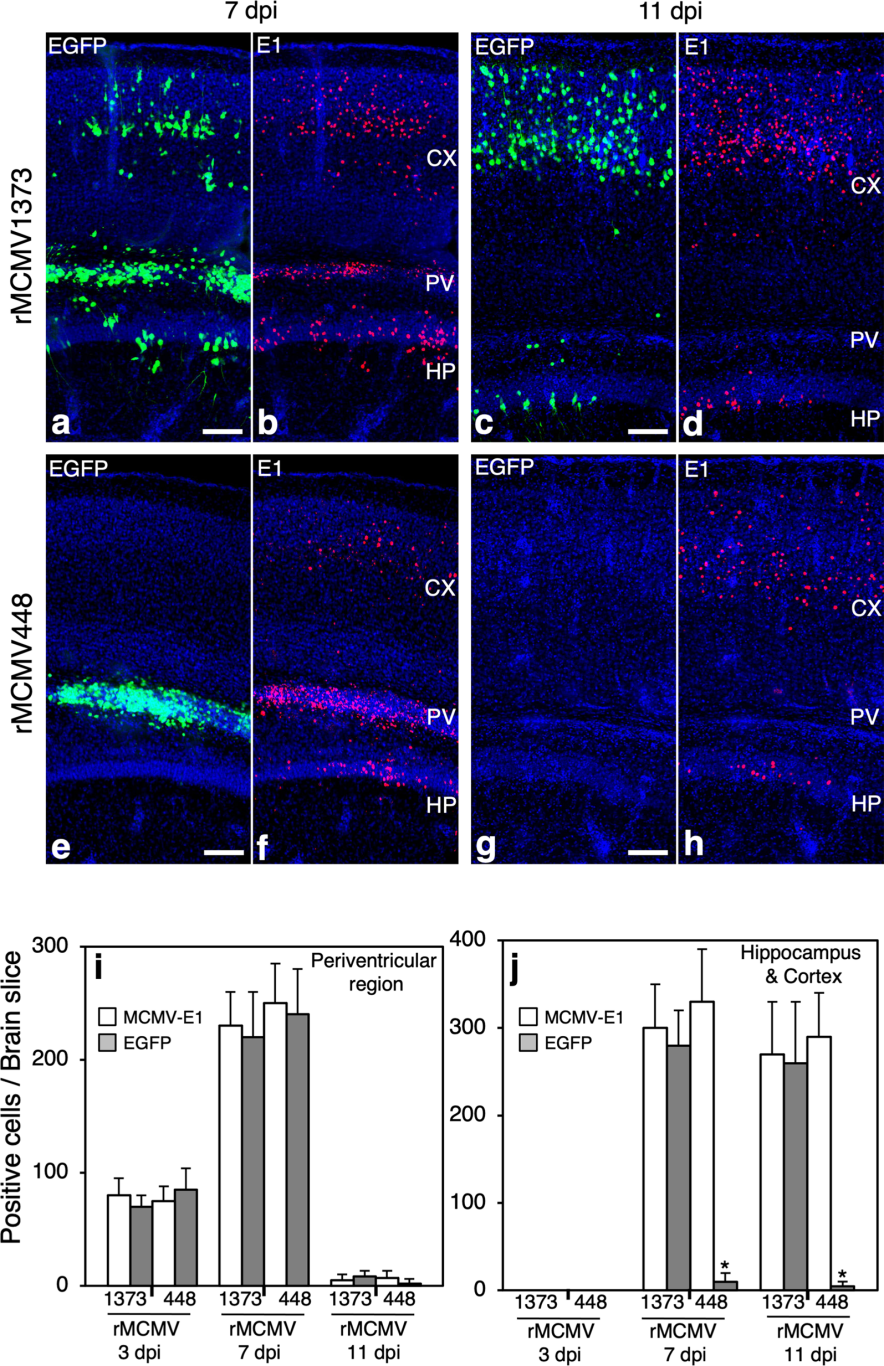
**a**–**h** Detection of EGFP and MCMV-E1 in neonatal mouse cerebrums infected with rMCMV1373 or rMCMV448 at 7 and 11 dpi. **a**–**d** rMCMV1373-infected cerebrums at 7 (**a**, **b**) and 11 dpi (**c**, **d**). **e**–**h** rMCMV448-infected cerebrum at 7 (**e**, **f**) and 11 dpi (**g**, **h**). In each rMCMV-infected cerebrum, fluorescence views of EGFP (**a**, **c**, **e**, **g**) and immunofluorescence for MCMV-E1 (red) (**b**, **d**, **f**, **h**) in the same field as the EGFP image are shown. Nuclei are stained with DAPI. CX, cortex; HP, hippocampus; PV, periventricular region. Scale bars: **a**–**h** 150 µm. **i**, **j** Kinetics of EGFP^+^ and MCMV-E1^+^ cells in the coronal sections of the right hemisphere of rMCMV-infected brains. Right hemispheres were divided into the PV, HP, and CX regions. The numbers of positive cells in each area at 3, 7, and 11dpi were counted and the numbers of positive cells in the hippocampal and cortical region were combined. **i** Kinetics of E1^+^ or EGFP^+^ cells in the PV region. **j** Kinetics of E1^+^ or EGFP^+^ cells in the HP and CX regions. Mean ± SEM of 3 mouse brains in each experimental group are shown (E1^+^ cells, white bar; EGFP^+^ cells, gray bar). *p < 0.01 vs. corresponding rMCMV1373-infected HP and CX in each experimental group by an unpaired 2-tailed Student’s *t*-test

At both 7 and 11 dpi in the rMCMV1373-infected cerebrums the localization of EGFP^+^ cells closely corresponded to that of E1^+^ cells (Fig. 7a–d). In either the PV region or the combined region of the HP and CX, there was no significant difference in cell numbers between EGFP^+^ cells and E1^+^ cells (Fig. 7i, j). In the PV region of the rMCMV448-infected cerebrums, the localization of EGFP^+^ cells closely corresponded to that of E1^+^ cells (Fig. 7e, f). At all time-points the numbers of EGFP^+^ cells were not significantly different from those of E1^+^ cells (Fig. 7i). On the contrary, at all time-points in the HP and CX of the rMCMV448-infected cerebrum EGFP^+^ cells were negligible (Fig. 7e, g) despite the presence of similar numbers of E1^+^ positive cells in the rMCMV1373-infected cerebrum (Fig. 7f, h, j).

These results suggest that *e1-pro*-448 is not activated in hippocampal or cortical neurons, but is sufficiently activated in the cells in the PV region and that the upstream region from nt -449 to -1373 in the *e1-pro*-1373 sequence is indispensable for the activation of *e1-pro* in hippocampal and cortical neurons.

#### Activation of MCMV e1-pro-1373 in neurons but not in astrocytes during the postnatal cerebral development

**Fig. 8:**
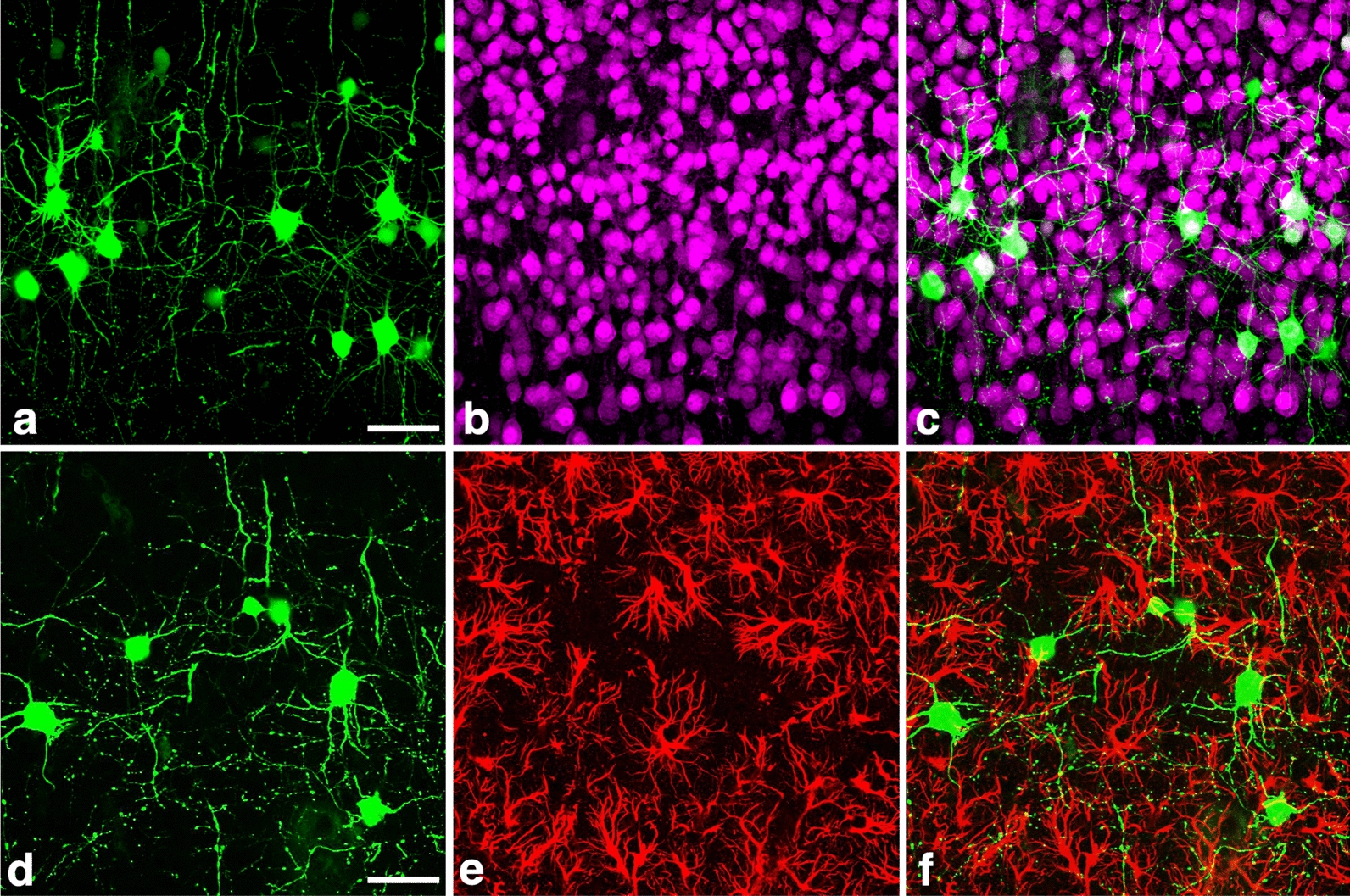
The activation of MCMV *e1-pro-*1373 exclusively in neurons, but not in differentiated CX astrocytes during the second postnatal week. Fluorescence views of EGFP (**a**, **d**), immunofluorescence for NeuN (**b**) or GFAP (**e**) and merged images of EGFP and each marker (**c**, **f**) in the CXs of neonatal mice infected with rMCMC1373 at 11dpi are shown. **a** Almost all EGFP^+^ cells demonstrate a morphology of the pyramidal neuron. **b** In the same field as **a**, NeuN (purple) is expressed in the nuclei of CX neurons. **c** A merged image of **a** and **b**. Nuclear areas of EGFP^+^-CX neurons appear whitish because of the co-localization of EGFP and NeuN, indicating that *e1-pro-*1373 is distinctly activated in neurons. **d** Another fluorescence image of EGFP. **e** In the same field as **d**, GFAP (red) is expressed in the cytoplasm of differentiated astrocytes, a morphology of which is distinctly different from that of EGFP^+^-pyramidal neurons in **d**. **f** A merged image of **d** and **e**. EGFP is not accurately co-localized with GFAP, indicating that the activation of *e1-pro-*1373 does not occur in differentiated CX astrocytes. Scale bars: **a**–**f** 50 µm

To clarify the exclusive neuronal activation of *e1-pro*-1373 during the postnatal cerebral development, the immunofluorescence for cellular markers was performed in rMCMV1373-infected cerebrums. At 7 dpi in the CX and HP, EGFP was expressed mostly in the neurons, though EGFP^+^ non-neuronal cells including vascular endothelial cell and perivascular macrophages/microglia were also found (data not shown). At 11 dpi these EGFP^+^ non-neuronal cells disappeared and EGFP was exclusively expressed in pyramidal neurons positive for NeuN (Fig. 8a–c). At all time-points in the CX and HP, no EGFP expression was observed in GFAP^+^ differentiated astrocytes (Fig. 8d–f).

These results clearly demonstrated that the upstream region from nt -449 to -1373 in the *e1-pro*-1373 sequence is necessary for the exclusive activation of *e1-pro* in cerebral neurons, particularly during the postnatal period from around 1 to 2 weeks (the second postnatal week). In addition, differentiated astrocytes are not permissive for MCMV infection as observed in vitro.

#### Expression of MCMV antigens and the activation of MCMV e1-pro-1373, but not -448, exclusively in cerebral neurons, particularly around the second postnatal week of cerebral development

**Fig. 9:**
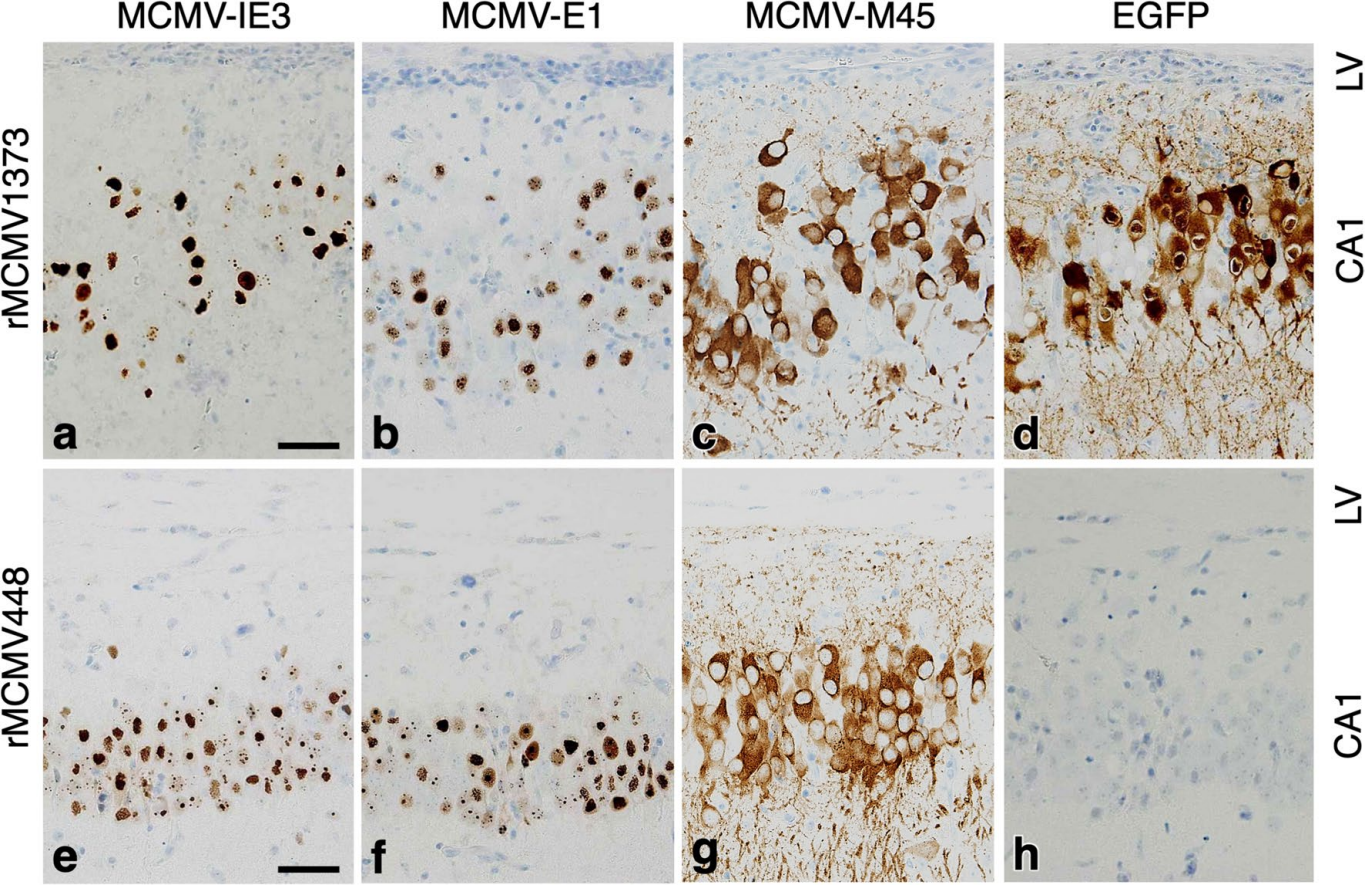
Immunohistochemical detection of MCMV-IE3, -E1, -M45 and EGFP in the serial sections of the HP infected with rMCMV1373 (**a**–**d**) or -448 (**e**–**h**) at 11 dpi. **a**, **e** MCMV-IE3 (brown) is expressed in the nuclei of the hippocampal neurons infected with either rMCMV1373 (**a**) or rMCMV448 (**e**). **b**, **f** MCMV-E1 (brown) is expressed in the nuclei in a similar manner to IE3. **c**, **g** MCMV-M45 (brown) is also expressed in the cytoplasm of hippocampal neurons with a pyramidal morphology. **d**,** h** EGFP (brown) was strongly expressed in the whole cell bodies of pyramidal neurons in the rMCMV1373-infected HP (**d**), but not expressed in the rMCMV448-infected HP (**h**). Nuclei are counterstained with hematoxylin. LV, lateral ventricle. Scale bars: **a**–**h** 100 µm

To precisely investigate the expression of MCMV antigens in addition to E1 and the activation of *e1-pro* in the cerebrum, particularly around the second postnatal week, immunohistochemical studies for MCMV-IE3, -E1, -M45 and EGFP were performed in the cerebrum infected with either rMCMV1373 or rMCMV448 at 11 dpi. MCMV-IE3, which is necessary to activate *e1-pro* [34, 39], was expressed to a similar level as E1 in the nuclei of hippocampal neurons of the rMCMV-infected cerebrum (Fig. 9a, b, e, f). MCMV-M45, which is known to inhibit necrosis of MCMV-infected cells [57], was also expressed in the cytoplasm of hippocampal neurons, preserving their pyramidal morphology (Fig. 9c, g). On the contrary, EGFP was not expressed in the rMCMV448-infected HP (Fig. 9h) despite sufficient expression of IE3, E1 and M45 (Fig. 9e–g), while in the rMCMV1373-infected HP, EGFP was strongly expressed in the whole cell body of pyramidal neurons (Fig. 9d).

These results confirm that the upstream region from nt -449 to -1373 in the *e1-pro*-1373 sequence is necessary for the neuron-specific activation of *e1-pro* associated with a unique persistent infection during the second postnatal week.

#### Transcription of MCMV-ie3, -e1 and EGFP mRNA in the cerebrums infected with wild type MCMV and rMCMVs

**Fig. 10:**
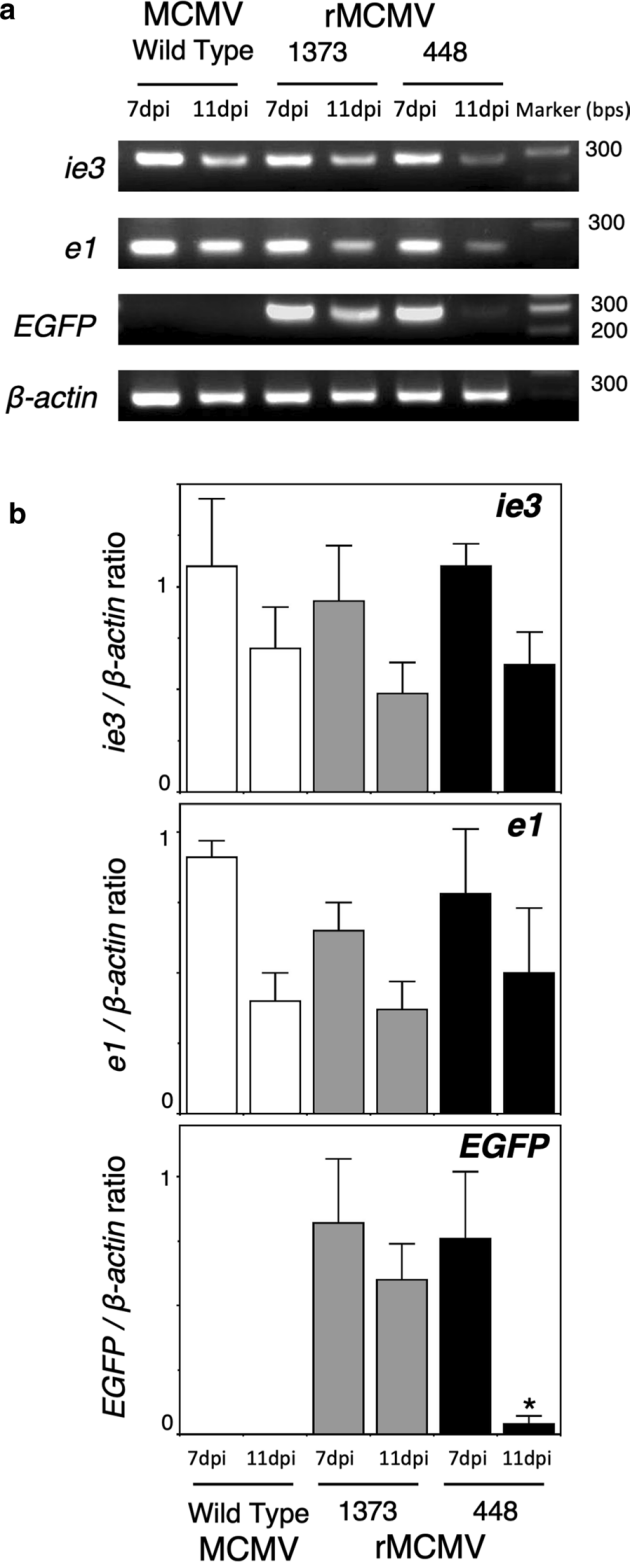
RT-PCR analyses for the transcription of MCMV-IE3, MCMV-E1 and EGFP mRNA in the cerebrums infected with wild-type MCMV and rMCMVs at 7 and 11 dpi. The RT-PCR product of constitutively expressed ß-actin was used as a control. **a** Representative data from three independent experiment are shown. **b** The ratio of the intensity of the RT-PCR signal of IE3, E1 or EGFP to that of ß-actin. Mean ± SEM of 3 mouse cerebrums in each experimental group are shown (wild-type MCMV, white bar; rMCMV1373, gray bar; rMCMV448, black bar). *p < 0.01 vs. corresponding rMCMV1373-infected cerebrum at 11 dpi by an unpaired 2-tailed Student’s *t*-test

We investigated the transcription of MCMV-E1 and EGFP mRNA in the cerebrums infected with wild type MCMV and rMCMVs by RT-PCR. In the cerebrums infected with any MCMVs, RT-PCR signals of the transcripts of IE3 and E1 mRNA were detected at both 7 and 11 dpi, although the transcriptions of IE3 and E1 mRNA at 11dpi were relatively lower than those at 7dpi (Fig. 10). This reduction in E1 mRNA transcription at 11dpi supported the results that viral growth and the number of E1^+^ cells in virus-infected cerebrums peaked around 7dpi and decreased at 11 dpi in any MCMV, as described above (Fig. 7, 8). On the contrary, the transcripts of EGFP mRNA in the rMCMV448-infected cerebrum disappeared at 11 dpi despite sufficient MCMV-E1 mRNA transcription.

Concerning the kinetics of EGFP expression in the virus-infected cerebrums (Fig. 7, 10), it is thought that EGFP mRNA transcription would be derived from both neurons and non-neuronal cells in the rMCMV1373-infected cerebrums. However, the transcripts in the rMCMV448-infected cerebrums were derived from non-neuronal cells. Furthermore, EGFP mRNA transcription in the rMCMV1373-infected cerebrums at 11dpi is considered to represent the almost complete neuron-specific transcription of EGFP. Therefore, these transcriptional analyses are consistent with the concept that the upstream region from nt -449 to -1373 in the *e1-pro*-1373 sequence is necessary for the exclusive activation of *e1-pro* in cerebral neurons, particularly during the second postnatal week of cerebral development.

## Discussion

The brain is the major target organ in congenital CMV infection. Although multiple mechanisms have been proposed to play potential roles in brain abnormalities induced by CMV, there are still unknown problems, especially in terms of long-term abnormalities [14]. The study of human subjects for congenital CMV infection has obvious limitations, particularly those of focusing on spaciotemporal analysis, and those on the brain as a whole, although cerebral organoid cultures have been developed using human materials [25]. We have studied the pathogenesis of the developmental disorders in mouse models using MCMV [55, 56]. MCMV shows similar characteristics to HCMV in terms of cell tropism, immunity in host–pathogen interactions [42] and genomic composition [35, 41].

The susceptibility to CMV and outcomes of brain abnormalities are closely related to not only the gestational age at infection but also the permissiveness of different brain cell types such as NSPCs, neurons, glial cells and macrophages [14]. Among these cell types, it was previously reported that the postnatal developing cerebral neurons in mice displayed a unique persistent infection with the expression of MCMV *e1* gene (M112-113) product (E1) [48]. Then, transgenic mice carrying the long *e1-pro*-*lacZ* reporter construct consistently demonstrated the *e1-pro* activation specifically in their neurons [3]. In this study we sought to generate the rMCMVs carrying constructs made up of different lengths of *e1-pro* connected with an EGFP reporter in order to determine the spaciotemporal activation of *e1-pro* during actual MCMV infection in vitro and in vivo. We have, for the first time, elucidated that the upstream sequence from nt -449 to -1373 in the longer *e1-pro*-1373 is necessary for the neuron-specific activation of *e1-pro* and the unique persistent infection in postnatal developing cerebral neurons, unlike the situation of non-neuronal cells.

It is possible that the deletion of the MCMV-M128 gene via the insertion of the *e1-pro* construct into the wild type MCMV genome causes rMCMVs to alter their viral nature, particularly in terms of pathogenesis. However, such a possibility is minimal for the following reasons. It has been reported that the deletion of the MCMV-M128 gene has no effect on viral growth in MEFs as well as on growth, latency, and pathogenesis in mice [11]. Furthermore, in this study, a comparison of rMCMVs with wild type MCMV revealed no significant differences in viral replication or E1 expression either in vitro or in vivo, suggesting that the recombination of the wild type virus has no effect on the pathogenesis or activation of *e1-pro* originally located in the viral genome.

During lytic infection, MCMV-E1-proteins, similarly to HCMV-E1-proteins, are essential for viral DNA replication, as they can bind DNA and accumulate in viral replication compartments within the host cell nuclei [1, 33, 45]. Eventually, the host cell dies after the production of viral progeny with rapid and exponential viral growth. This kind of infection is exactly observed in CMV infections of MEFs and NSPCs [28, 32, 37, 44]. In CMV-infected embryonic NSPCs from the PV region, the disruption of their proliferation and differentiation causes serious and permanent sequelae such as microcephaly [31, 51]. Furthermore, the activation of *e1-pro* by MCMV-IE3 (product of *ie* gene; M122-123), a homologue of HCMV-IE2, is critical for the expression of E1-proteins [34, 39]. It is thought that IE3 can interact with *e1-pro* and TATA binding protein (TBP) simultaneously to clamp the transcription factor IID (TFIID) complex onto *e1-pro*, which drives *e1* gene transcription [1, 33, 39, 45]. Specific binding sites of MCMV-IE3 or HCMV-IE2 are known to exist between the transcriptional start site and nt -172 or nt -290, respectively, within the CMV *e1-pro* sequences [4, 39, 47]. The present study consistently confirmed that the short *e1-pro*-448 including the IE3 binding site is sufficient to activate E1 expression and cause lytic infection in non-neuronal cells such as MEFs, NSPCs and perivascular macrophages in the developing brain. Most previous reports concerning CMV infection of the developing brain also demonstrated that lytic CMV infection occurred, particularly in the PV region either in animal models [13, 29, 31, 44] or human materials [51].

Unlike the lytic infection of non-neuronal cells, MCMV-infected neurons, either in primary neuronal cultures or postnatal brains, demonstrated a unique persistent infection. These neurons, accounting for 90% of MCMV-infected cells in primary neuronal cultures, showed very a low and flat viral titer of less than one thousandth of that in MCMV-infected MEFs, although these neurons adequately expressed E1 as observed in virus-infected MEFs. Similarly, in postnatal mice brains infected with MCMV, cerebral neurons of the HP and CX at 11 dpi exclusively showed prolonged expression of E1, despite a marked reduction in viral titer in the brain and the disappearance of lytic infection in the PV region. Interestingly, the activation of the short *e1-pro*-448 in MCMV-infected neurons did not occur entirely in vitro or in vivo, though IE3 is properly expressed in these neurons and the long *e1-pro*-1373 was sufficiently activated. These results indicate that the short *e1-pro*-448 sequence alone is insufficient to drive the IE3-mediated activation of *e1-pro* in neurons or that it undergoes certain inhibition unlike in non-neuronal cells. Thus, the upstream sequence from nt -449 to -1373 in the long *e1-pro*-1373 sequence is supposed to work as an enhancer that is necessary to activate *e1-pro,* particularly in MCMV-infected neurons.

The transgenic mice carrying a long *e1-pro*, such as *e1-pro*-1373, demonstrated *e1-pro* activation specifically in the postnatal cerebral neurons without MCMV infection or activation via IE3-binding, suggesting that the long *e1-pro* including the upstream enhancer sequence on the host genome could be independently activated through a unique mechanism exclusive to these neurons [3]. Furthermore, because of the augmentation of *e1-pro* activation by MCMV infection in the cerebral neurons of the transgenic mice, the upstream enhancer would interact with neuron-specific factor(s) to support *e1-pro* activation via IE3 binding. Therefore, during actual MCMV infection the upstream enhancer sequence may be essential in driving the neuron-specific activation of *e1-pro*.

In the developing neurons it is possible that the activation of *e1-pro* at peaks during the postnatal period from around 1 to 2 weeks (the second postnatal week), according to the following studies as well as the present one. Previous reports concerning intrauterine MCMV infection demonstrated that immature neurons in the embryonic cerebrum during corticogenesis did not express MCMV-E1, despite NSPCs showing lytic infection [31, 44]. However, at P7 (13 dpi), MCMV-E1 was expressed in the cortical and hippocampal neurons [48]. Thereafter MCMV-E1 expression in neurons disappeared until postnatal 1 month [52]. Similarly, in postnatal MCMV infection, E1 was not expressed in the cerebral neurons during the early postnatal phase. Then, at P8, MCMV-E1 was observed to dramatically appear in the cerebral neurons in the present and previous studies [26, 29]. This unique expression of MCMV-E1 in the cerebral neurons was still maintained at P12 and thereafter gradually declined (data not shown). These findings suggest that in the immature neurons during the embryonic and early postnatal phase, MCMV-E1 may not be expressed due to the complete repression of *e1-pro* activity. Then, around the second postnatal week, this repression may be removed and, at the same time, *e1-pro* activated. It is supposed that the upstream enhancer sequence participates in the removal of this repression specifically in the developing neurons at this time.

During the second postnatal week in rodents which corresponds to the human gestational period from around 19 to 24 weeks [16], the cortical pyramidal neurons demonstrate rapid growth in overall dendrite length, more than doubling in a week [30, 43]. Furthermore, the cerebrum extensively reorganizes gene expression for completing neuronal differentiation and promoting the processes of maturation such as synaptogenesis, neurite outgrowth and myelination [36, 50]. It is possible that in MCMV-infected developing neurons these substantial transcriptional changes would also trigger the removal of the repression of *e1-pro* activation through the probable action of the upstream enhancer sequence. In addition, it is conceivable that MCMV proteins including E1, which are induced after the release of the repression, may adversely impact on the cellular functions of the developing cerebral neurons, resulting in the neurodevelopmental disorders. This concept seems to be supported by previous reports that CMV infection in the hippocampal neurons reduces the expression of the N-methyl-d-aspartate (NMDA) subtype of glutamate receptors, which are essential for the plasticity of synapses and the learning [18, 26, 60].

It has been reported that many neurotropic viruses cause persistent neuronal infection without cytopathicity [19, 38]. Although in MCMV-infected neurons the expression of viral antigens including E1 seems to persist until no longer than postnatal 1 month [52], it is expected that these neurons could survive with certain functional disturbances caused by MCMV infection, as described above, for the following reasons. First, MCMV-infected neurons both in vitro and in vivo preserved fine neuronal morphology without cytopathic effects, such as signs of lytic cell death observed in non-neuronal cells. In these neurons, the level of MCMV gene expression may be restricted so that lytic cell death with the production of infectious viral particles would hardly occur. Furthermore, these neurons are considered to resist cell death, such as excitotoxic cell death, induced by excess glutamate [27], because MCMV has several genes for anti-apoptosis and -necrosis proteins, including M45, as shown in this study [9, 27, 57]. Second, it is well-known that CMV has strategies for the evasion of innate immunity [20]. During MCMV infection of the neonatal brain, MCMV-infected cerebral neurons preferentially escaped the detection of NK cells and macrophages, and survived, while non-neuronal cells with lytic viral infection in the PV region were attacked by these innate immune cells and disappeared [29]. Interestingly, persistent HCMV infection in vitro was also demonstrated in human neurons differentiated from neonatal neural progenitor cells. These neurons survived with the preservation of neuronal morphology and displayed the persistent expression of viral antigens without cytopathic effects [32]. Therefore, these dysfunctional surviving neurons caused by CMV infection may be responsible for the subsequent neurodevelopmental disorders including psychiatric diseases.

In recent years, it has been reported that perinatal insults to the developing brain such as infections, inflammation or immunological disturbance appear to cause neuropsychiatric consequences in later life, such as autism spectrum disorders or schizophrenia [2, 6, 23]. In the case of HCMV infection, the linking of perinatal HCMV infection to schizophrenia has been controversial [7, 12], while Børglum et al. reported a significant relationship between maternal HCMV infection and some populations of schizophrenia patients [8]. However, until now there have been no human studies with direct evidence of HCMV infection in the cerebral neurons of patients with these neuropsychiatric diseases.

Hence, our finding of a unique neuron-specific MCMV infection in the postnatal stage of murine cerebral development may provide a clue to investigating the pathogenesis of neuropsychiatric diseases.

## Conclusions

In this study we tried to clarify factors that determine the susceptibility of developing cerebral neurons to CMV infection. Based on an established mouse model of MCMV infection, we focused on the mechanism of promoter activation of an essential viral gene, MCMV-*e1* gene, in developing cerebral neurons of neonatal mice. By means of the rMCMVs carrying constructs made up of different lengths of *e1-pro* connected with an EGFP reporter, the spaciotemporal activation of *e1-pro* during actual MCMV infection in vitro and in vivo were investigated. Consequently, we found that short *e1-pro*-448 is sufficient to activate E1 expression in non-neuronal cells, however, the upstream sequence from nt -449 to -1373 in long *e1-pro*-1373 is supposed to work as an enhancer necessary for the neuron-specific activation of *e1-pro* associated with a persistent infection, particularly around the second postnatal week. This unique activation of *e1-pro* in developing cerebral neurons may be an important factor in the neurodevelopmental disorders induced by congenital CMV infection.

## Supplementary Information

**Additional file 1: Figure S1.** The detailed construction, preparation method and verification of rMCMVs. **a** Construction of rMCMV448 and rMCMV1373. Map of the *Hind*III F, K, L and J fragments of the MCMV (Smith strain) genome. The arrangements of the original MCMV-*e1-pro* (position 161605 to 162977), M112/113 (*e1*) (position 1612978 to 165076), M122/123 (*ie*1/*ie*3) and M128 genes (position 186085 to 187296) in wild type MCMV are shown. During MCMV infection, IE3 protein, which is translated from the spliced transcript of the M122/123 gene, binds and trans-activates MCMV-*e1-pro*, causing E1-protein production. In this study, an *e1-pro*-EGFP cassette consisting of an *e1-pro* fragment (nt -1373 or -448 to + 38 relative to the transcription start site) (black arrow), EGFP gene (gray arrow) and SV40-derived polyadenylation signal (short black box), was inserted into the position between the 5′- (183081 to 184430) and 3′- (187159 to 188573) flanking sequences (striped arrows) in the MCMV genome by homologous recombination. This recombination causes the deletion of the nt 2728 sequence including the greater part of the M128 gene (184431 to 187158). However, the M128 gene is completely dispensable for viral growth in cell cultures as well as for growth, latency, and pathogenesis in mice [11]. It is supposed that the deletion of the M128 gene has almost no effect on endogenous *ie* promoter activation. During the infection of recombinant viruses, the activation of the inserted *e1-pro* can be in situ detected as the expression of EGFP (in situ reporter assay in Fig. S1b). **b** A recomobinant virus was created by the co-transfection of MEFs with the genomic DNA of MCMV Smith strain and a recombinant DNA fragment of the *e1-pro*-EGFP cassette with the 5′-(position from 183078 to 184442) and 3′-(position from 187159 to 188573) flanking sequences using FuGENE 6 transfection reagent (Promega, #E2691, Madison, WI). A transfer vector, which contained the flanking DNA fragment described above, was constructed based on the plasmid vector pEGFP-C1 (Gene accession number U55763, Clontech, Takara Bio USA). After the deletion of the HCMV enhancer/promoter sequence and multiple cloning sites from pEGFP-C1, the PCR-amplified *e1-pro*, 5′-, and 3′-flanking sequences were cloned into the *Ase*I/*Nhe*I, *Ase*I and *Mlu*I sites of pEGFP-C1, respectively. During the PCR amplification of the flanking sequences, novel *Kpn*I sites were made at the start of the 5′- and the end of the 3′-flanking sequences, so that a recombinant DNA fragment of the *e1-pro*-EGFP cassette with 5′- and 3′-flanking sequences was readily prepared by *Kpn*I-digestion of the transfer vector. After the co-transfection of MEFs with viral genomic DNA and a recombinant DNA fragment as described above, the cells in a plaque with green fluorescence foci were picked up and transferred to an uninfected cell culture. This plaque purification was done at least three times. A virus preparation was considered pure when all foci resulting from limiting-dilution infections with this preparation displayed EGFP expression, indicating that no wild-type virus remained in the preparation. **c** Verification of rMCMV DNA. Viral genomic DNA was purified from virus-infected cells according to the Hirt method [Hirt B (1967) Selective extraction of polyoma DNA from infected mouse cell cultures. J Mol Biol 26:365–369. https://doi.org/10.1016/0022-2836(67)90307-5]. Inserted sequences between the 3′-end of the 5′-flanking sequence and the 3′-end of the *e1-pro*-EGFP cassette were amplified by PCR using 5′-primer (GTC TTA TGG GTA GGG GGC TT) and 3′-primer (ATG AGT TTG GAC AAA CCA CAA C). To determine the size of the inserted *e1-pro* sequences, PCR products were digested by *Nhe*I. On the other hand, the deletion of the M128 gene from the rMCMV DNA was examined by PCR amplification of a specific DNA sequence (186919 to 187125) in the M128 gene using 5′-primer (CTG AAG GAC AGG GTG TTC GT) and 3′-primer (AGC TAG CCT CCT CAC CTT CC). Inserted sequences between the 3′-end of the 5′-flanking sequence (5′-primer, arrow head) and the 3′-end of the *e1-pro*-EGFP cassette (3′-primer, arrow head) were specifically amplified as 2.4 kbp and 1.5 kbp signals from rMCMV-1373 and -448 DNA, respectively (lane 3 and 4), but not from the wild type MCMV DNA (lane 2). After the *Nhe*I digestion of amplified sequences, 1.4 kbp and 0.5 kbp products of the expected promoter sequences were determined in rMCMV1373 and -448 DNA, respectively (lane 5 and 6, arrow heads). On the other hand, the 207 bp signal of the specific DNA sequence (186919 to 187125) in the M128 gene was identified in the wild type MCMV DNA (lane 8), but not in the rMCMV-1373 or -448 DNA (lane 9 and 10). These results indicate that the *e1-pro*-EGFP cassettes were accurately inserted into the expected position in the viral genomic DNA. Furthermore, the inserted *e1-pro*-EGFP cassettes amplified by PCR using the 5′- and 3′-primers described above (lane 3 and 4) were sequenced and confirmed as appropriate inserts (Advanced Research Facilities and Services, Hamamatsu University School of Medicine).

## Data Availability

All data used and analyzed for the current study are available from the corresponding author on reasonable request.

## Abbreviations

MCMV: Murine cytomegalovirus
HCMV: Human cytomegalovirus
*e1*: Early gene 1
E1: Early protein 1
*ie3*: Immediate-early gene 3
IE3: Immediate-early protein 3
*e1-pro*: *e1*-Promoter
EGFP: Enhanced green fluorescent protein
rMCMV: Recombinant MCMV
MEF: Mouse embryonic fibroblast
NSPC: Neural stem/progenitor cells
PV: Periventricular
HP: Hippocampus
CX: Cerebral cortex
MOI: Multiplicity of infection
PFU: Plaque forming unit
dpi: Days post-infection
