# Supplementary figures and images for: Prolonged activation of cytomegalovirus early gene e1-promoter exclusively in neurons during infection of the developing cerebrum
Source: Acta Neuropathol Commun. 2021 Mar 9;9:39. doi: 10.1186/s40478-021-01139-0 (PMC7941713; doi:10.1186/s40478-021-01139-0)

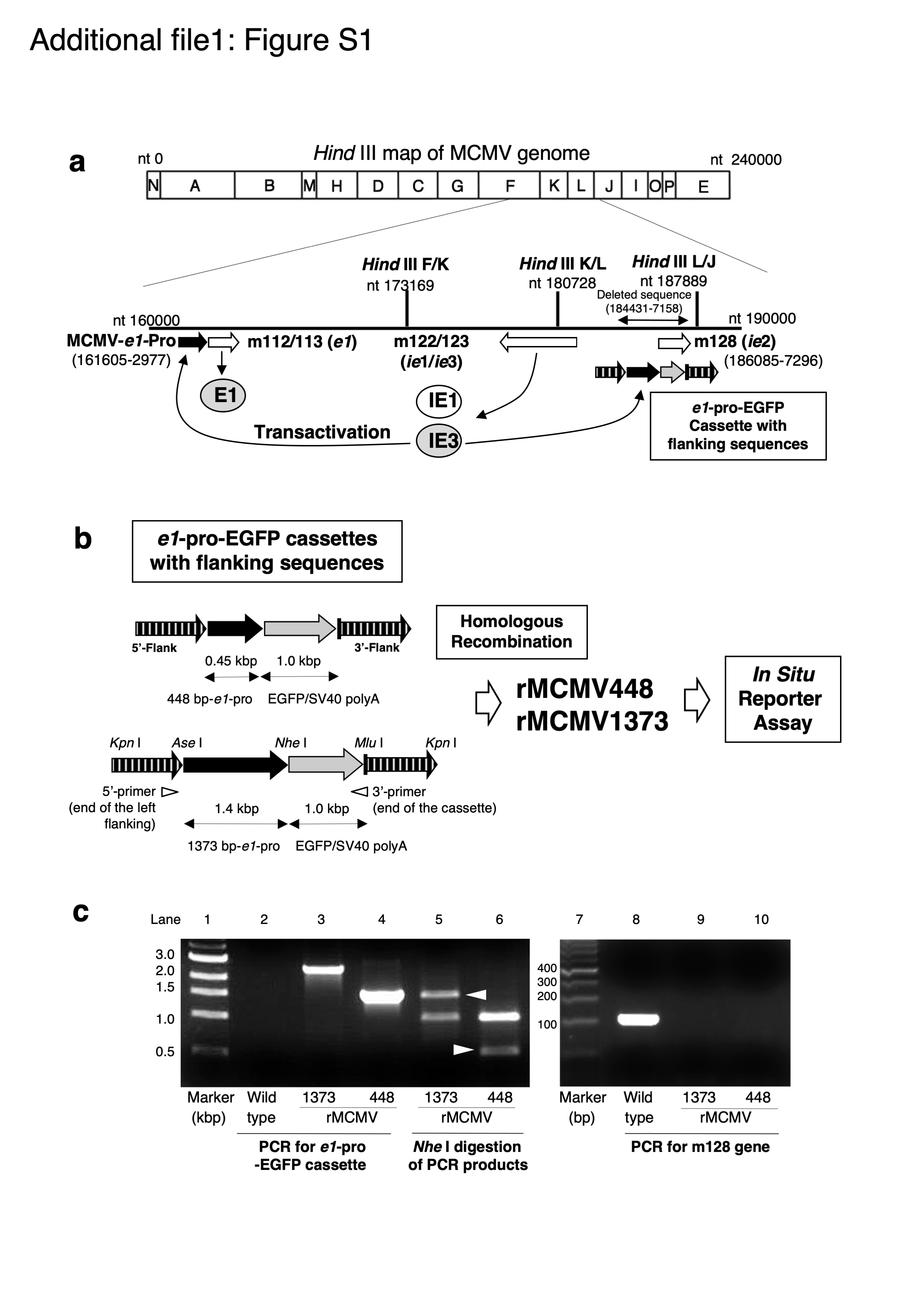

Supplement: Supplementary file 1 — Additional file 1: Figure S1. The detailed construction, preparation method and verification of rMCMVs. a Construction of rMCMV448 and rMCMV1373. Map of the HindIII F, K, L and J fragments of the MCMV (Smith strain) genome. The arrangements of the original MCMV-e1-pro (position 161605 to 162977), M112/113 (e1) (position 1612978 to 165076), M122/123 (ie1/ie3) and M128 genes (position 186085 to 187296) in wild type MCMV are shown. During MCMV infection, IE3 protein, which is translated from the spliced transcript of the M122/123 gene, binds and trans-activates MCMV-e1-pro, causing E1-protein production. In this study, an e1-pro-EGFP cassette consisting of an e1-pro fragment (nt -1373 or -448 to + 38 relative to the transcription start site) (black arrow), EGFP gene (gray arrow) and SV40-derived polyadenylation signal (short black box), was inserted into the position between the 5′- (183081 to 184430) and 3′- (187159 to 188573) flanking sequences (striped arrows) in the MCMV genome by homologous recombination. This recombination causes the deletion of the nt 2728 sequence including the greater part of the M128 gene (184431 to 187158). However, the M128 gene is completely dispensable for viral growth in cell cultures as well as for growth, latency, and pathogenesis in mice [11]. It is supposed that the deletion of the M128 gene has almost no effect on endogenous ie promoter activation. During the infection of recombinant viruses, the activation of the inserted e1-pro can be in situ detected as the expression of EGFP (in situ reporter assay in Fig. S1b). b A recomobinant virus was created by the co-transfection of MEFs with the genomic DNA of MCMV Smith strain and a recombinant DNA fragment of the e1-pro-EGFP cassette with the 5′-(position from 183078 to 184442) and 3′-(position from 187159 to 188573) flanking sequences using FuGENE 6 transfection reagent (Promega, #E2691, Madison, WI). A transfer vector, which contained the flanking DNA fragment described above, was con [file 40478_2021_1139_MOESM1_ESM.tif]
